# Giant Thoracic Aortic Aneurysm Rupture in a Patient with Extensive Atherosclerotic Disease

**DOI:** 10.5334/jbsr.3314

**Published:** 2024-02-02

**Authors:** Ramona Mihaela Popa, Chiriță Andrei-Constantin, Rosana Mihaela Manea

**Affiliations:** 1 Department of Radiology and Medical Imaging, Clinical Emergency County Hospital of Brașov, 500326 Brașov, Romania; 2 Department of Radiology and Medical Imaging, Clinical Emergency County Hospital of Brașov, 500326 Brașov, Romania; 3 Faculty of Medicine, “Transilvania” University of Brașov, Romania, 500036 Brașov, Romania, Department of Radiology and Medical Imaging, Clinical Emergency County Hospital of Brașov, 500326 Brașov, Romania

**Keywords:** aorta, thoracic aortic aneurysm, thoracic aortic aneurysm rupture, atherosclerotic disease, CT angiography

## Abstract

Aneurysmal dilatations can affect any aortic segment and represent the result of various causes, atherosclerotic disease being the most common and frequently involved.

We hereby illustrate a case of a patient with thoracic aortic aneurysm rupture due to extensive atherosclerotic disease, with multiple complex penetrating ulcerated atherosclerotic plaques located in the descending aorta.

CT angiography evaluation included a comprehensive description of imaging features and extent of the thoracic aortic aneurysm, the presence of thrombus, relationship to adjacent structures and branches, associated complications.

*Teaching Point:* Thoracic aortic aneurysm rupture due to extensive atherosclerotic disease with multiple penetrating ulcers.

## Case History

A 69-year-old male was admitted to the Emergency Department with persistent cough, haemoptysis, haematemesis, dyspnoea and low blood pressure. The patient’s history includes grade 3 hypertension and chronic kidney disease stage V.

**Figure 1:**
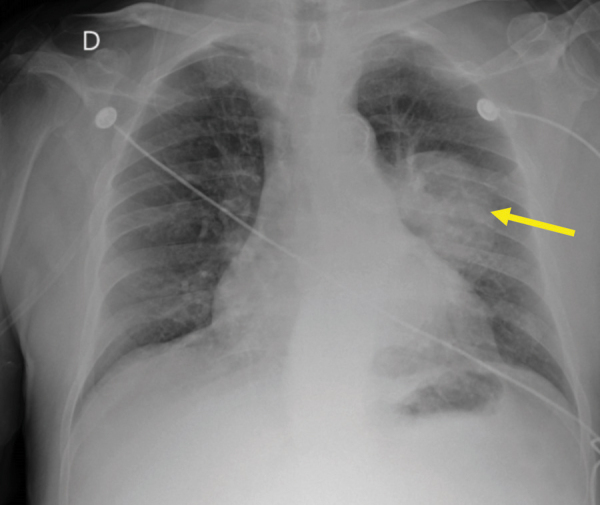
Frontal chest X-ray.

A frontal chest X-ray (Figure 1) illustrated a left perihilar well-defined macronodular opacity of medium intensity (yellow arrow). Bilateral interstitial densification with a reticular pattern and diffuse distribution can be noted.

**Figure 2:**
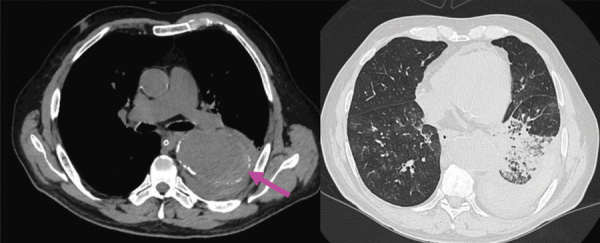
Native CT acquisitions - axial sections.

Native CT acquisitions (Figure 2) depicted a large descending aortic calibre with a discrete hyperattenuating crescentic sign on the left lateral aspect of the descending aorta (pink arrow). Left pleural effusion, with hyperdense areas, suggestive of haemothorax, can be noted.

Multiple pulmonary consolidations in the left inferior lobe and micronodular interstitial densification patterns in the right lower lobe can be noted—suggestive of intrapulmonary haemorrhage, cannot exclude the coexistence of an infectious component.

**Figure 3:**
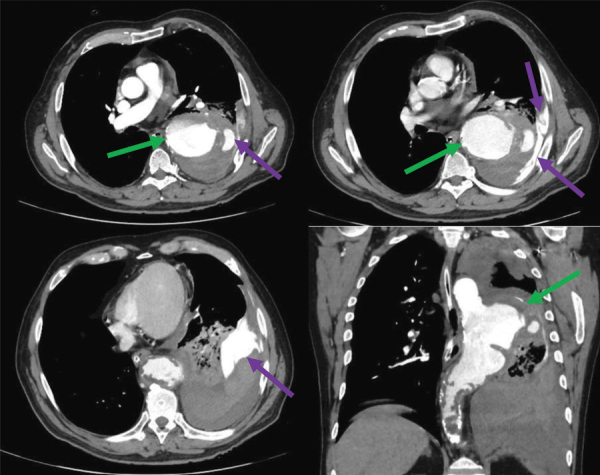
Contrast-enhanced CT acquisitions - arterial phase - axial and coronal sections.

**Figure 4:**
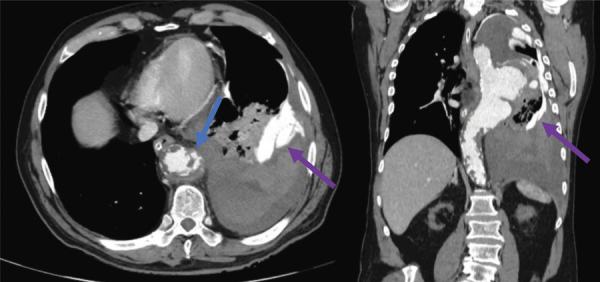
Contrast-enhanced CT acquisitions - arterial phase - axial and coronal sections.

Contrast-enhanced CT acquisitions (Figures 3 and 4) revealed rupture of a giant saccular aneurysm of the descending thoracic aorta (green arrows) with active contrast substance extravasation into the left inferior pulmonary lobe and into the left pleural cavity with associated haemothorax (purple arrows).

The aortic aneurysm measured 55/67 mm in the axial plane and 104 mm in the cranio-caudal length. The proximal aspect was projected near the aortic arch (vertebral level T4–T5) at 90 mm caudal to the left subclavian artery emergence, and the distal extent was projected at T9 vertebral level. An associated circumferential irregular mural thrombus can be noted.

The entire aorta and all its branches demonstrate remarkably widespread atherosclerosis, including multiple complex plaques. Multiple atherosclerotic non-calcified plaques with irregular margins pointing to the aortic lumen, most of them ulcerated, can be seen in the descending aortic segment (Figure 4—blue arrow).

## Comment

An aneurysm represents an abnormal focal dilatation of a blood vessel [1]. Thoracic aortic aneurysms are divided into true aneurysms and false aneurysms (pseudoaneurysms) [1]. True aneurysms contain all three layers of the aortic wall, whereas false aneurysms never involve all three layers and are contained by the adventitia or periadventitial tissues [1].

Thoracic aorta aneurysmal dilatations are mostly the result of atherosclerotic disease, as illustrated in our case [1]. Other causes include degeneration of the medial layer of the aortic wall (idiopathically or due to genetic disorders, including Marfan syndrome and Ehlers–Danlos syndrome), aortic dissection, trauma, syphilis, noninfective aortitis, rheumatic fever, rheumatoid arthritis, ankylosing spondylitis, giant cell arteritis, relapsing polychondritis, Takayasu arteritis, Reiter syndrome, systemic lupus erythematosus, scleroderma, psoriasis, ulcerative colitis, radiation, Behçet disease and congenital anomaly [1].

Initial evaluation includes a CT angiography of the entire thoraco-abdominal aorta, because an abdominal aortic aneurysm occurs in 28% of patients with a thoracic aortic aneurysm [1].

In our case, the aortic aneurysm rupture was the result of an extensive atherosclerotic disease with multiple penetrating ulcers in the descending aorta.

The most important differential diagnosis should always include aortitis, which presents concentric regular wall thickening and hypoattenuating wall, which enhances postcontrast!
